# Cerebral Lesions in Pregnancy and Parturition

**Published:** 1907-09

**Authors:** Walter C. Swayne

**Affiliations:** Professor of Midwifery, University College, Bristol; Obstetric Physician, Bristol Royal Infirmary.


### CEREBRAL LESIONS IN PREGNANCY AND PARTURITION.

BY

Walter C. Swayne, M.D., B.S.,

*Professor of Midwifery, University College, Bristol; Obstetric Physician, Bristol Royal Infirmary.*

---

Organic cerebral lesions may occur in the course of pregnancy and parturition with the same relative frequency as in the absence of these conditions, but their coincidence may, under certain circumstances, give rise to considerable difficulties in diagnosis.

In addition to symptoms due to organic lesions of the brain and cord, several forms of neurosis may occur which simulate them in a greater or less degree, and give rise to difficulties in diagnosis and treatment which may lead to mistakes, both in treatment and prognosis, of a character by no means trivial.

The position is also complicated by the fact that various functional neuroses, with symptoms superficailly resembling those of organic cerebral lesions, are liable to occur during both pregnancy and the puerperal state, *e.g.* affections of speech, apparent loss or impairment of vision, affections of hearing and paretic conditions are met with not infrequently; while inflammatory cerebral lesions, with their accompanying symptoms in the puerperium, will almost certainly be attributed in the first instance to uterine sepsis, with which, it must be stated, there is occasionally a definite causal connection. Paralyses of the lower limbs alone are not infrequent, owing to the liability of the nerve tissues in the pelvis to mechanical pressure; these may be attributed to lesions of the cord, and it is not unknown for cases superficially or even intimately resembling, *e.g.*, spastic paraplegia to arise, or at any rate to display their first symptoms after parturition. The writer has quite recently met with a case of this kind, in which paraplegic symptoms with spastic phenomena supervened on a difficult labour, but proved on careful investigation not to be due to a definite descending spinal lesion, the symptoms of which they much resembled.

Such cases as this do not come within the purview of this paper, which is concerned more especially with cases in which the symptoms indicated an organic cerebral lesion.

**Case 1.**—A multipara, æt. 37, was delivered by a midwife, who summoned the writer on the day following delivery because the patient was paralysed. I found her to be suffering from marked right-sided hemiplegia, with interference with speech. She was quite unable to articulate distinctly; although she would make attempts to pronounce the words she wished to use, she could not do so in such a way as to make them recognisable. Some facial paralysis was present. The whole condition cleared up in about ten days, the paralysis passing off and her power of articulation returning. The lesion was regarded as being due to a thrombosis of the left middle cerebral artery or one of its branches.

**Case 2.**—A multipara, æt. 22, was delivered on the 27th of January, 1901, labour being normal in every respect. Two days later, during which time she had suffered from severe headache and rise of temperature, which was attributed to septic infection, and for which her uterus was washed out, she became comatose, and died very shortly afterwards. An autopsy was made, and to the surprise of everyone concerned pus was found over the whole upper surface of the cerebral hemispheres and anterior third of the cerebellum; also between the lobes of the cerebellum and along the whole length of the cord. Numerous cocci were found in the pus, including a diplococcus which stained feebly with Gram. The cause of death was obviously suppurative cerebro-spinal meningitis.

**Case 3.**—A patient who was at about the end of the seventh month of pregnancy was admitted into the Bristol Royal Infirmary in a state of coma. She had some slight convulsive movements, her urine was albuminous, temperature 102° F., pulse 110, and respirations 50; a little later her temperature was 103° F., pulse 110, and respirations 72, the coma increasing. She was wet-cupped at once, and the usual remedies for eclampsia used in the interval between her admission and my seeing her. On seeing her, I expressed a doubt as to her suffering from eclampsia, and hazarded the suggestion that she was in reality suffering from an inflammatory cerebral lesion, possibly meningitis; but of this there were no definite symptoms, except that her general aspect and the character of the convulsive movements reminded me of the appearance of children suffering from meningitis. She delivered herself twenty hours later of a premature stillborn child, and died four hours later. *Post-mortem:* Suppurative meningitis of the brain and cord was found. The pus contained numerous staphylococci, and in addition a diplococcus resembling Fraenkel's.

**Case 4.**—A multipara, aged about 40, was admitted in a state of coma, with convulsive movements and albuminous urine, as suffering from eclampsia. She was in the ninth month of pregnancy, and was delivered by *accouchment forcée* but died within two hours of delivery. *Post-mortem:* There was found at the upper part of the right temporal lobe a cyst filled with fluid as big as the fist, and a second cyst of less size was found in the lower right frontal lobe. The kidneys were granular.

**Case 5.**—A multipara, aged 28, on the tenth day after delivery developed a temperature of 103° F., after a convulsive seizure, which was chiefly marked on the left side; her temperature gradually rose, and she rapidly became comatose, but no more fits occurred. When I saw her there was an occasional tremor of the left arm and leg, associated with marked rigidity of these limbs, deviation of the eyes to the right, and some facial paralysis. She had suffered from a discharge from the right ear. She died about eight hours later, and no autopsy was made. This was probably a case of cerebral abscess with rupture following on middle ear disease.

From a practical point of view, the importance of these cases resolves itself into considerations of diagnosis and prognosis, since in the last case only could treatment have had any marked effect. In Case 1 there was no great difficulty, except that either the hemiplegia, affection of speech, or facial paralysis might, if they had existed alone, been possibly functional.

The presence of these three conditions simultaneously, however, appeared to me to indicate a gross cerebral lesion, while the comparatively rapid recovery and the fact that the paralyses and affection of speech were not absolute seemed to me to point to a thrombosis rather than an embolus or hemorrhage.

In Case 2 the only clue to the real source of the symptoms lay in the acute headache and ingravescent coma, with high fever.

In such a case one's first thought would be naturally that uterine sepsis was the cause, the only possibility of excluding which would be to prove that the uterus was germ-free by culture. Cerebro-spinal meningitis was never dreamt of as a possible cause of the symptoms, or a lumbar puncture would in all probability have cleared up the diagnosis. One point in connection with this case is worth recording : in the majority of cases in which suppurative cerebro-spinal meningitis has been found as a complication of the puerperium uterine sepsis has been present.

This, after all, is not surprising; but the rapid death in this case almost precludes that possibility, while at the autopsy no focus of septic infection was found. There is no record as to whether a culture was taken from the uterine cavity.

The infection must almost certainly have been present at the time of delivery, as the patient was complaining of headache then.

In the third case a quite justifiable diagnosis of eclampsia was made. I did not agree with this, in spite of the albuminous urine, because the so-called fits in no way resembled eclamptic seizures as I have seen them, and, moreover, in eclampsia a markedly febrile temperature does not as a rule occur except after several fits, and when it does is of very grave prognostic import.

The rapid respirations rather suggested pneumonia, but no physical signs could be found.

None of the usual symptoms attributed to meningitis were present in any noticeable degree, and I greatly regret that, having hazarded the suggestion that cerebro-spinal meningitis was the cause of the trouble, I did not at once proceed to verify this by lumbar puncture.

In Case 4 the fits were probably eclamptic, and the cysts present simply a coincidence.

In Case 5 the febrile temperature, increasing coma, left-sided spasm and rigidity rather indicated the presence of a localised cerebral inflammatory process than an attack of eclampsia, which was the provisional primary diagnosis. In this case possibly surgical intervention might have succeeded had her condition been such as to call the attention of her medical attendant or to alarm her friends earlier. As it was, her own attendant was not sent for until she was obviously very ill, and when I saw her she was moribund.

*Prognosis.*— In any case similar to those mentioned above prognosis cannot be too guarded if a gross cerebral lesion is probable, and especially if it appears to be of an inflammatory origin. Whether in connection with pregnancy or not, this may be looked upon as the rule.

A mistake in diagnosis is quite likely, and in the majority of such cases quite pardonable, even with a practitioner of experience. In the cases of meningitis it is extremely doubtful if an accurate diagnosis would have been of the least value.

It is barely possible that the injection of an antiseptic through the lumbar puncture might have some effect ; in any case it could hardly hasten the certain fatal event ; or it is again possible that injection of a polyvalent serum might prove of some use, or at any rate of little harm. The rapid fatality of the affection gives but little time for anything of the kind, however, and in these cases one important point is to be remembered—that whatever is to be done should be carefully and clearly explained to the friends, who are particularly prone to misinterpret unsuccessful efforts in these cases, and who are most obstinate and unreasonable in sticking to their own opinion as to the cause of death.

As an example of this, I may mention a case of which I have known, in which the patient dying of cerebral hemorrhage shortly after a confinement, in which she was given chloroform while forceps were used, the anæsthetic has always been spoken of by the friends as the cause of death, and the reputation of its administrator made to suffer accordingly.

### REFERENCES.

**Galabin**—*Midwifery*, 1904, p. 875.

**Edgar**—*Obstetrics*, 1903, p. 361.

**Boislinière**—*Obstetric Accidents, Emergencies, and Operations*, 1896, p. 160.

*System of Gynæcology and Obstetrics*. Ed. by Mann and Hirst. Vol. ii. part ii., 1889, p. 497.

**Imbert-Gourbeyre,** quoted by Hirst. *Ibid.*, p. 627.

**Norris and Dickinson**—*Text-book of Obstetrics*, vol. ii., 1902, p 157.

